# A population‐based meta‐analysis of circulating GFAP for cognition and dementia risk

**DOI:** 10.1002/acn3.51652

**Published:** 2022-09-03

**Authors:** Mitzi M. Gonzales, Crystal Wiedner, Chen‐Pin Wang, Qianqian Liu, Joshua C. Bis, Zhiguang Li, Jayandra J. Himali, Saptaparni Ghosh, Emy A. Thomas, Danielle M. Parent, Tiffany F. Kautz, Matthew P. Pase, Hugo J. Aparicio, Luc Djoussé, Kenneth J. Mukamal, Bruce M. Psaty, William T. Longstreth, Thomas H. Mosley, Vilmundur Gudnason, Djass Mbangdadji, Oscar L. Lopez, Kristine Yaffe, Stephen Sidney, R. Nick Bryan, Ilya M. Nasrallah, Charles S. DeCarli, Alexa S. Beiser, Lenore J. Launer, Myriam Fornage, Russell P. Tracy, Sudha Seshadri, Claudia L. Satizabal

**Affiliations:** ^1^ Glenn Biggs Institute for Alzheimer's & Neurodegenerative Diseases University of Texas Health Science Center at San Antonio San Antonio Texas USA; ^2^ Department of Neurology University of Texas Health Science Center at San Antonio San Antonio Texas USA; ^3^ Department of Population Health Sciences University of Texas Health Science Center at San Antonio San Antonio Texas USA; ^4^ South Texas Veterans Health Care System, Geriatric Research Education & Clinical Center San Antonio Texas USA; ^5^ Cardiovascular Health Research Unit University of Washington Seattle Washington USA; ^6^ Laboratory of Epidemiology and Population Sciences, Intramural Research Program National Institute on Aging Bethesda Maryland USA; ^7^ The Framingham Heart Study Framingham Massachusetts USA; ^8^ Department of Neurology Boston University School of Medicine Boston Massachusetts USA; ^9^ Department of Biostatistics Boston University School of Medicine Boston Massachusetts USA; ^10^ Brown Foundation of Molecular Medicine, McGovern Medical School University of Texas Health Science Center at Houston Houston Texas USA; ^11^ Department of Pathology and Laboratory Medicine, and Biochemistry, Larner College of Medicine University of Vermont Burlington Vermont USA; ^12^ School of Psychological Sciences, Turner Institute for Brain and Mental Health Monash University Clayton Victoria Australia; ^13^ Harvard T.H. Chan School of Public Health Boston Massachusetts USA; ^14^ Department of Medicine Brigham and Women's Hospital Boston Massachusetts USA; ^15^ Boston Veterans Affairs Healthcare System Boston Massachusetts USA; ^16^ Department of Medicine Beth Israel Deaconess Medical Center Boston Massachusetts USA; ^17^ Department of Epidemiology University of Washington Seattle Washington USA; ^18^ Department of Medicine University of Washington Seattle Washington USA; ^19^ Department of Health Systems and Population Health University of Washington Seattle Washington USA; ^20^ Department of Neurology University of Washington Seattle Washington USA; ^21^ The MIND Center University of Mississippi Medical Center Jackson Mississippi USA; ^22^ Icelandic Heart Association Research Institute Kópavogur Iceland; ^23^ Department of Cardiology University of Iceland Reykjavik Iceland; ^24^ Department of Neurology University of Pennsylvania Philadelphia Pennsylvania USA; ^25^ Department of Psychiatry University of California San Francisco California USA; ^26^ Department of Neurology University of California San Francisco California USA; ^27^ Department of Epidemiology and Biostatistics University of California San Francisco California USA; ^28^ San Francisco VA Medical Center San Francisco California USA; ^29^ Kaiser Permanente Medical Center Program Oakland California USA; ^30^ Department of Radiology University of Pennsylvania Philadelphia Pennsylvania USA; ^31^ Department of Neurology University of California Davis California USA

## Abstract

**Objective:**

Expression of glial fibrillary acidic protein (GFAP), a marker of reactive astrocytosis, colocalizes with neuropathology in the brain. Blood levels of GFAP have been associated with cognitive decline and dementia status. However, further examinations at a population‐based level are necessary to broaden generalizability to community settings.

**Methods:**

Circulating GFAP levels were assayed using a Simoa HD‐1 analyzer in 4338 adults without prevalent dementia from four longitudinal community‐based cohort studies. The associations between GFAP levels with general cognition, total brain volume, and hippocampal volume were evaluated with separate linear regression models in each cohort with adjustment for age, sex, education, race, diabetes, systolic blood pressure, antihypertensive medication, body mass index, apolipoprotein E ε4 status, site, and time between GFAP blood draw and the outcome. Associations with incident all‐cause and Alzheimer's disease dementia were evaluated with adjusted Cox proportional hazard models. Meta‐analysis was performed on the estimates derived from each cohort using random‐effects models.

**Results:**

Meta‐analyses indicated that higher circulating GFAP associated with lower general cognition (*ß* = −0.09, [95% confidence interval [CI]: −0.15 to −0.03], *p* = 0.005), but not with total brain or hippocampal volume (*p* > 0.05). However, each standard deviation unit increase in log‐transformed GFAP levels was significantly associated with a 2.5‐fold higher risk of incident all‐cause dementia (Hazard Ratio [HR]: 2.47 (95% CI: 1.52–4.01)) and Alzheimer's disease dementia (HR: 2.54 [95% CI: 1.42–4.53]) over up to 15‐years of follow‐up.

**Interpretation:**

Results support the potential role of circulating GFAP levels for aiding dementia risk prediction and improving clinical trial stratification in community settings.

## Introduction

The past decade has seen tremendous progress in the validation of biological markers for Alzheimer's disease and related dementias (ADRD), even extending into the pre‐symptomatic stage.^1^, ^2^ Amyloid and tau positron emission tomography (PET) imaging have the capacity to detect abnormal protein deposition 15 to 20 years prior to clinical diagnosis.^3^ While PET imaging has provided major advances within clinical and research settings, a need still exists for inexpensive, minimally invasive, and broadly available screening tools.^4^ With the use of ultrasensitive assays, the hallmark biological features of Alzheimer's disease (AD), including amyloid beta 40 and 42 and phosphorylated tau 217 and 181, can be reliably detected in blood.^5^, ^6^

Blood‐based assays also afford the opportunity to simultaneously evaluate proteins reflecting diverse pathophysiological processes underlying ADRD, which may facilitate identification of new drug targets and precision medicine approaches.^7^ Even as a singular diagnostic entity, AD is highly heterogenous with numerous pathways implicated beyond amyloid beta and tau deposition.^8^ Furthermore, individuals with dementia often present with multiple co‐pathologies at autopsy,^9^ highlighting the need for broader screening approaches. While the combination of elevated cerebral amyloid beta and tau is considered specific for AD, glial dysfunction and neuroinflammation manifest across dementia subtypes.^10^ Growing research supports the fundamental role of reactive astrocytosis in neurodegenerative disease with elevated glial fibrillary acidic protein (GFAP) expression as a primary marker.^11^ GFAP expression is increased in the brains of individuals with AD, often colocalizing with plaques and tangles.^12^, ^13^ Cerebrospinal fluid (CSF) levels have been shown to differentiate individuals with dementia from cognitively unimpaired adults.^14^, ^15^ With the use of ultrasensitive assays, GFAP levels can be detected in blood, and some recent studies have reported that circulating levels associate with poorer cognition and ADRD status.^16^, ^17^, ^18^, ^19^, ^20^ Relevant to secondary prevention efforts, plasma GFAP levels have also been found to predict amyloid positivity among cognitively unimpaired adults.^21^, ^22^, ^23^, ^24^ Despite these encouraging findings, substantial heterogeneity exists. A recent meta‐analysis reported higher CSF GFAP levels in individuals with AD relative to cognitively unimpaired adults.^15^ However, plasma GFAP levels failed to distinguish between groups. Methodological factors, including use of varied assays, may have contributed to the heterogeneity in outcomes.^25^ Thus, the need is pressing for further validation of circulating GFAP in longitudinal, population‐based cohort studies that can help extend generalizability to community settings.

The goal of the present study was to examine the associations between circulating GFAP levels with cognition, total brain and hippocampal volume, and incident all‐cause and AD dementia. Meta‐analysis was conducted across four longitudinal population‐based cohort studies, the Framingham Heart Study (FHS), the Cardiovascular Health Study (CHS), the Age, Gene/Environment Susceptibility – Reykjavik Study (AGES), and the Coronary Artery Risk Development in Young Adults (CARDIA) Study with previously reported harmonized cognitive, neuroimaging, and clinical outcomes.^26^, ^27^, ^28^, ^29^ For the current study, circulating GFAP levels were assessed using the same ultrasensitive assay platform across cohorts to reduce heterogeneity. Based on previous research,^18^, ^19^, ^20^ we hypothesized that higher GFAP levels would associate with poorer cognition, smaller total brain and hippocampal volume, and increased risk of incident dementia.

## Methods

### Study samples

#### FHS

The FHS is a community‐based, single site prospective cohort spanning three generations of participants from Framingham, Massachusetts.^30^ The Original Cohort was established in 1948 and their descendants, alongside their spouses, were offered enrollment in the Offspring Cohort, beginning in 1971. The Offspring Cohort participants have completed up to nine quadrennial examinations. Beginning in 1994, Framingham residents between the ages of 40 and 75 years who identified as a member of a diverse ethnic or racial group were recruited from the community to establish the Omni Cohort. The Omni Cohort members have completed up to four examinations in parallel with the Offspring Cohort. Plasma from fasting blood draws performed at examination 9 in the Offspring Cohort (2011–2014) and examination 4 in the Omni Cohort (2011–2014) were used to assay GFAP.

#### CHS

CHS was established in 1989 as an observational cohort study of community‐dwelling adults, aged 65 years and older, across Forsyth County, North Carolina; Washington County, Maryland; Sacramento County, California; and Pittsburgh, Pennsylvania.^31^ Between 1992 to 1993, the cohort was complemented by the inclusion of 687 mostly Black Americans. CHS participants have completed up to ten annual examinations. Serum from fasting blood drawn at the Year 9 examination (1996–1997) was used to assay GFAP. Eligibility for participation in the Year 9 examination included individuals who were free of treated diabetes.

#### AGES

AGES is a prospective, single‐site cohort study of residents of Reykjavik, Iceland, which was established in 1967 by the Icelandic Heart Association.^32^ In 2002, the surviving members of the cohort, who were aged 67 years or older, were invited for re‐examination, which included the collection of plasma from fasting blood draws that was used to assay GFAP.

#### CARDIA

The CARDIA Study is a multisite population‐based study conducted across Birmingham, Alabama; Chicago, Illinois; Minneapolis, Minnesota; and Oakland, California. The study was established by the National Heart, Lung, and Blood Institute in 1984.^33^ Black and White adults, between the ages of 18 to 30 years at the time of enrollment, were recruited. Participants have completed up to 9 examinations over 30 years. Plasma from fasting blood drawn at the Year 25 examination (2010–2011) was used to assay GFAP.

#### Standard protocols and approvals

Institutional review boards at each enrolling institution approved all studies, and participants provided written informed consent prior to enrollment. To be eligible for the current study, participants had to have data on circulating GFAP levels and had to lack prevalent dementia at the time of the blood draw used for assays. There were no additional exclusion criteria.

#### Quantification of circulating GFAP levels

Across cohorts, fasting blood samples were centrifuged, aliquoted, and stored at −80 degrees Celsius. Blood specimens were assayed for GFAP using Simoa Neurology 4‐Plex E kits and a Simoa HD‐1 Analyzer (Quanterix, Lexington, MA, catalog #102153) at the Laboratory for Clinical Biochemistry Research at The University of Vermont. Prior research has demonstrated excellent convergence between GFAP levels obtained in plasma and serum using the Simoa platform with comparisons yielding non‐significant results.^25^ The analytical range was between 4.64 and 3784 pg/mL, and the mean interassay coefficient of variance was 9.70%. The assays were performed by a certified laboratory‐technician blinded to demographic and clinical data.

#### Cognitive assessments

Cognitive assessments administered within 6 years of the blood draw for GFAP were included in the analyses. The four cohorts employed distinct cognitive batteries.

The FHS cognitive battery included Weschler Memory Scale (WMS) Logical Memory and Visual Reproduction Immediate and Delayed Recall, Trail Making Test Part B, and Similarities.^34^ CHS included the Modified Mini‐Mental Status Examination, Benson Visual Retention Test, and the Digit Symbol Substitution Test (DSST).^35^ In AGES, the cognitive measures included the Mini Mental Status Examination, Cambridge Neuropsychological Test Automated Battery Spatial Working Memory Task, Digit Span Backwards, Stroop Word Naming, Color Naming, and Color‐Word Interference, the California Verbal Learning Test Immediate and Delayed Free Recall, DSST, and the Figure Comparison Test.^36^ Cognitive assessments in CARDIA included the Rey Auditory Verbal Learning Test Immediate and Delayed Recall, the DSST, and the Stroop Inhibition score.^37^ As previously described,^26^ a standardized general cognition score was created in each cohort by conducting a prinicipal components analysis of the cognitive tasks following conversion to z‐scores. The general cognition score was derived from the first unrotated principal component (PC1).^26^ The PC1 variable was standardized to a z‐score with higher values indicating better performance.

#### Brain magnetic resonance imaging

The analysis included brain Magnetic Resonance Imaging (MRI) scans that were conducted within 6 years of the blood draw used to assay GFAP. Details on MRI parameters, processing, and harmonization across cohorts have been previously described.^27^ Briefly, total brain volume and intracranial volume (ICV) were derived using automated or semi‐automated post‐processing software. Hippocampal volumetry was quantified using manually‐defined boundaries drawn on serial coronal sections or using automated methods.^38^ MRI metrics were expressed as a percentage of ICV.

#### Ascertainment of incident dementia

As previously described, incident all‐cause and AD dementia were ascertained independently by each study through ongoing surveillance in the FHS,^39^ CHS,^40^ and AGES.^41^ Briefly, all‐cause dementia was adjudicated using criteria from the Diagnostic and Statistical Manual of Mental Disorders, 4th Edition (DSM‐IV).^42^ AD dementia was adjudicated based on the criteria of the National Institute of Neurological and Communicative Disorders and Stroke and the AD and Related Disorders Association (NINCDS‐ADRDA) for possible or probable AD.^43^

#### Statistical analysis

GFAP values were right‐skewed and were normalized using a natural log transformation. The logarithm transformed GFAP values were then standardized prior to analyses. Descriptive statistics were used to describe the demographic and clinical characteristics of each cohort. The associations between circulating GFAP levels with general cognition (PC1), total brain volume, and hippocampal volume were evaluated in each cohort using separate linear regression models adjusting for age, sex, education, race, diabetes, systolic blood pressure, antihypertensive medication use, body mass index, apolipoprotein E ε4 status (at least one ε4 allele vs. none), site (if a multi‐site study), and the time interval between the blood draw for GFAP and the outcome variable. In effort to aid validation in diverse cohorts, stratified analyses by race (White and Black) were performed in the two cohorts where sample size permitted, CHS and CARDIA. The associations between circulating GFAP levels and incident all‐cause and AD dementia were examined in FHS, CHS, and AGES among participants aged 60 or older using separate Cox proportional hazard models over a maximum of 15‐year follow‐up with age as the time‐scale and adjustment for sex, education, race, diabetes, systolic blood pressure, antihypertensive medication use, body mass index, apolipoprotein ε4 status (at least one ε4 allele vs. none), and site (if a multi‐site study). To account for the possibility of reverse causation, Cox proportional hazard models were repeated with dementia surveillance beginning 2 years after the blood draw for GFAP. The delayed surveillance models were only conducted in FHS and CHS as few cases in AGES occurred within the first 2 years. Stratified analyses by race were also performed for incident all‐cause and Alzheimer's disease dementia in CHS. For all non‐stratified models described above, meta‐analyses were performed on the estimates derived from each cohort using random‐effects models with the inverse variance method used to determine the weight of each study. The Sidik‐Jonkman estimator was used to report a robust overall association across studies.^44^ The meta‐analyses were performed using the package meta Version 4.18–1 and function metagen on RStudio Version 1.4.1106. All statistical tests were two‐sided and *p*‐values <0.05 were considered significant.

## Results

### Circulating GFAP and general cognition

**Figure 1:**
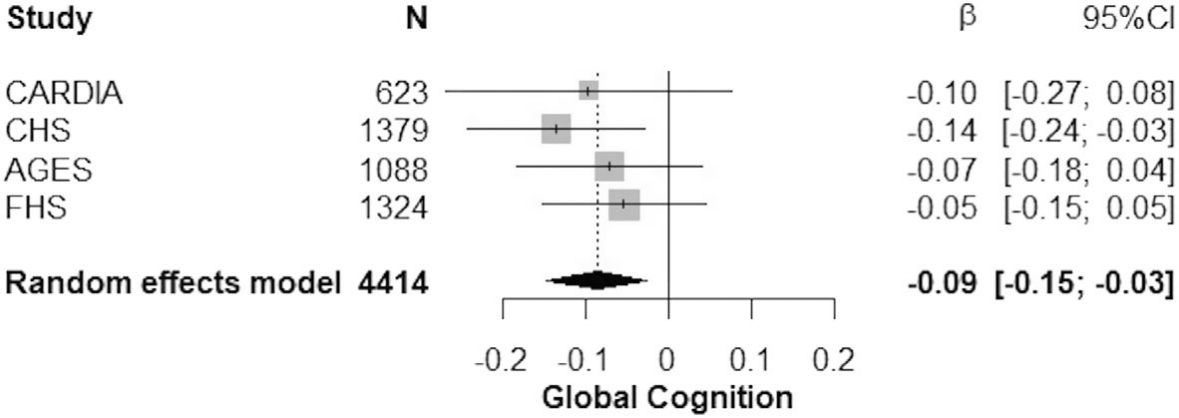
Pooled associations between circulating GFAP and general cognition. Results are per unit increase in the standardized natural log of GFAP. Linear regression models adjust for age, sex, education, race, diabetes, systolic blood pressure, antihypertensive medication use, body mass index, apolipoprotein E ε4 status (at least one ε4 allele vs. none), site (if a multi‐site study), and the time interval between the blood draw for GFAP and the outcome variable.

Demographics at the time of blood draw for GFAP within the sample used to examine associations with general cognition are presented in Table 1. The average time between the blood draw for GFAP and cognitive assessment ranged from 0 to 3 years across cohorts. The association between blood‐derived GFAP levels and general cognition only reached statistical significance in CHS, although the direction of effect was consistent across all cohorts (Fig. 1). Meta‐analysis results indicated that higher circulating GFAP was associated with lower general cognition. For each one standard deviation unit (SDU) increase in log‐transformed blood‐derived GFAP level, the general cognition score (PC1) declined by 0.09 SDUs. Results across cohorts were homogenous (*I* ^2^ < 0.001, *p* = 0.73).

**Table 1:** Cohort demographics – cognitive sample.

	FHS *N* = 1246	CHS *N* = 1379	AGES *N* = 1088	CARDIA *N* = 623
Age, years, mean (range)	69 (44–95)	77 (68–93)	76 (66–93)	50 (42–56)
Female, *n* (%)	706 (57%)	835 (61%)	610 (56%)	319 (51%)
Education, *n*, %				
Less than high school degree	33 (3%)	230 (17%)	256 (24%)	5 (<1%)
High school degree	264 (21%)	392 (28%)	532 (49%)	128 (21%)
Some college	374 (30%)	357 (26%)	179 (16%)	363 (58%)
College degree or higher	575 (46%)	396 (29%)	121 (11%)	127 (20%)
Race				
Black, *n* (%)	11 (<1%)	167 (12%)	0 (0%)	241 (39%)
White, *n* (%)	1122 (90%)	1212 (88%)	1088 (100%)	384 (61%)
Other, *n* (%)	113 (9%)	0 (0%)	0 (0%)	0 (0%)
Body mass index, m/kg^2^	28 ± 5	27 ± 4	27 ± 4	29 ± 6
Systolic blood pressure, mmHg	125 ± 16	136 ± 20	142 ± 20	117 ± 14
Antihypertensive medication, *n* (%)	666 (53%)	707 (51%)	696 (64%)	141 (23%)
Diabetes, *n* (%)	197 (16%)	32 (2%)	117 (11%)	63 (10%)
Presence of APOE ε4 Allele, No. (%)	287 (23%)	326 (24%)	297 (27%)	190 (30%)
Blood‐derived GFAP levels, pg/mL, median (Q1‐Q3)	168 (119, 240)	235 (179, 327)	176 (130, 232)	96 (73, 124)
Time from blood draw to cognitive assessment, years, mean (range)	2 (0–6)	2 (2–4)	0 (0)	0 (0)

All values represent mean ± standard deviation unless otherwise noted. AGES, Age, Gene/Environment Susceptibility – Reykjavik Study; APOE, Apolipoprotein E; CARDIA, Coronary Artery Risk Development in Young Adults Study; CHS, Cardiovascular Health Study; FHS, Framingham Heart Study; GFAP, Glial Fibrillary Acidic Protein; Q, Quartile.

### Circulating GFAP and neuroimaging outcomes

**Figure 2:**
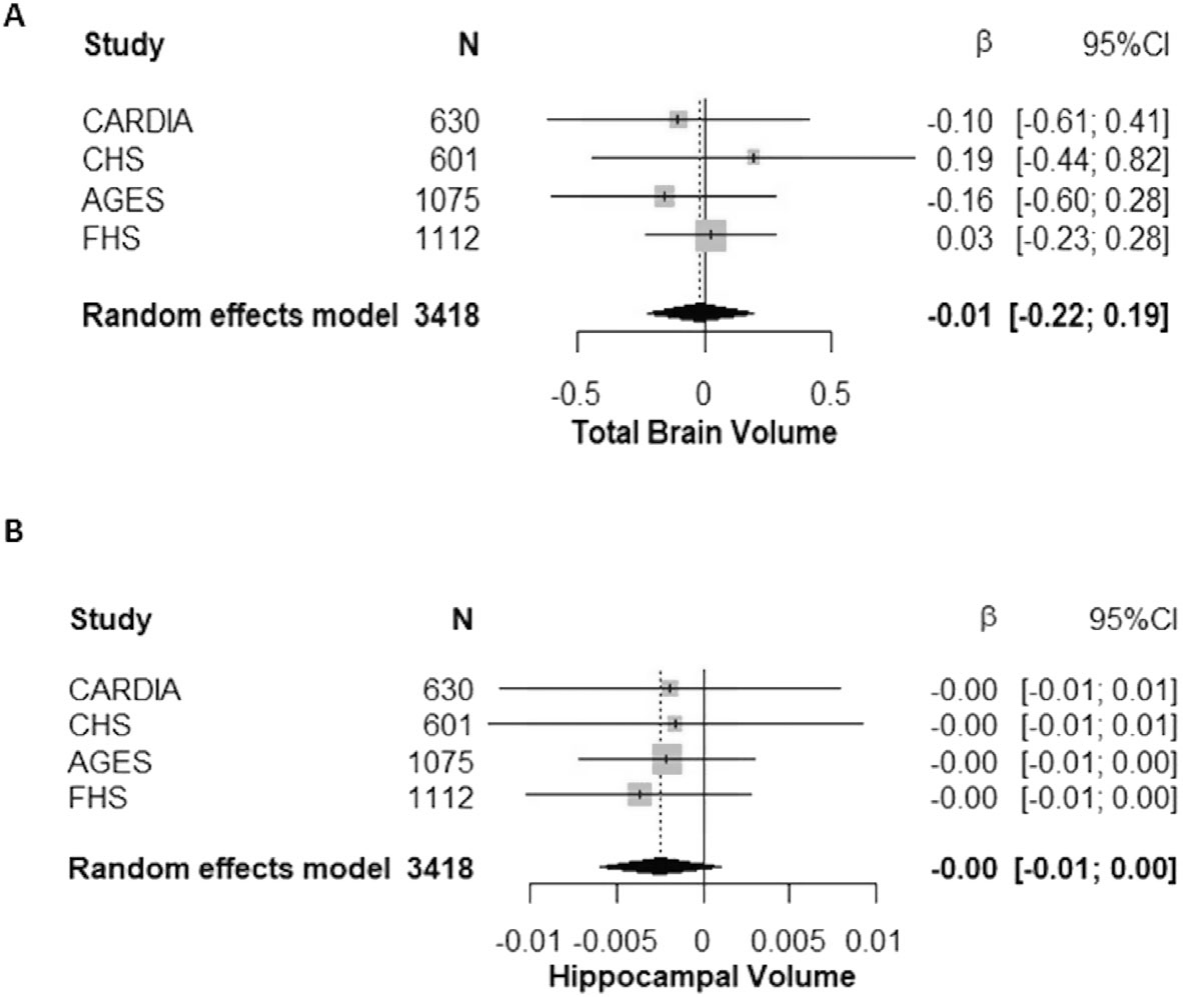
Pooled associations between circulating GFAP and neuroimaging outcomes. Results are per unit increase in the standardized natural log of GFAP examining associations with (A) total brain volume and (B) hippocampal volume. Linear regression models adjust for age, sex, education, race, diabetes, systolic blood pressure, antihypertensive medication use, body mass index, apolipoprotein E ε4 status (at least one ε4 allele vs. none), site (if a multi‐site study), and the time interval between the blood draw for GFAP and the outcome variable.

Descriptions of the neuroimaging sample are displayed in Table 2. The average time between the blood draw for GFAP and MRI ranged from 0 to 2 years across cohorts. There were no significant associations between GFAP with total brain or hippocampal volume across the individual cohorts. No significant associations were observed between GFAP with total brain or hippocampal volume in the meta‐analyses (Fig. 2A, Fig 2B). Results across cohorts were homogenous (total brain volume: *I* ^2^ < 0.001, *p* = 0.79, hippocampal volume: *I* ^2^ < 0.001, *p* = 0.98).

**Table 2:** Cohort demographics – neuroimaging sample.

	FHS *N* = 1112	CHS *N* = 601	AGES *N* = 1075	CARDIA *N* = 630
Age, years, mean (range)	69 (46–96)	77 (69–92)	76 (66–93)	50 (42–56)
Female, *n* (%)	618 (56%)	354 (59%)	602 (56%)	329 (52%)
Education, *n*, %				
Less than high school degree	32 (3%)	90 (15%)	247 (23%)	5 (<1%)
High school degree	241 (22%)	157 (26%)	527 (49%)	132 (21%)
Some college	312 (28%)	163 (27%)	172 (16%)	365 (58%)
College degree or higher	527 (47%)	191 (32%)	129 (12%)	128 (20%)
Race				
Black, *n* (%)	11 (<1%)	58 (10%)	0 (0%)	244 (39%)
White, *n* (%)	996 (90%)	541 (90%)	1075 (100%)	386 (61%)
Other, *n* (%)	105 (10%)	2 (<1%)	0 (0%)	0 (0%)
Body mass index, m/kg^2^	28 ± 5	26 ± 4	27 ± 4	29 ± 6
Systolic blood pressure, mmHg	125 ± 16	135 ± 20	142 ± 20	117 ± 14
Antihypertensive medication, *n* (%)	580 (52%)	266 (48%)	689 (64%)	146 (23%)
Diabetes, *n* (%)	172 (15%)	14 (2%)	111 (10%)	62 (10%)
Presence of APOE ε4 Allele, No. (%)	263 (24%)	139 (23%)	289 (27%)	193 (31%)
Blood‐derived GFAP levels, pg/mL, median (Q1–Q3)	168 (121, 242)	243 (176, 351)	177 (130, 232)	96 (74,125)
Time from blood draw to MRI, years, mean (range)	2 (0–5)	1 (0–3)	0 (0)	0 (0)
Total brain volumetry, percentage of intracranial volume	75.3 ± 2.5	67.7 ± 3.6	72.2 ± 3.8	85.2 + 2.8
Hippocampal volumetry, percentage of intracranial volume	0.53 ± 0.05	0.49 ± 0.06	0.37 ± 0.04	0.56 + 0.05

All values represent mean ± standard deviation unless otherwise noted. AGES, Age, Gene/Environment Susceptibility – Reykjavik Study; APOE, Apolipoprotein E; CARDIA, Coronary Artery Risk Development in Young Adults Study; CHS, Cardiovascular Health Study; FHS, Framingham Heart Study; GFAP, Glial Fibrillary Acidic Protein; Q, Quartile, MRI, Magnetic Resonance Imaging.

### Circulating GFAP and incident all‐cause and AD dementia

**Figure 3:**
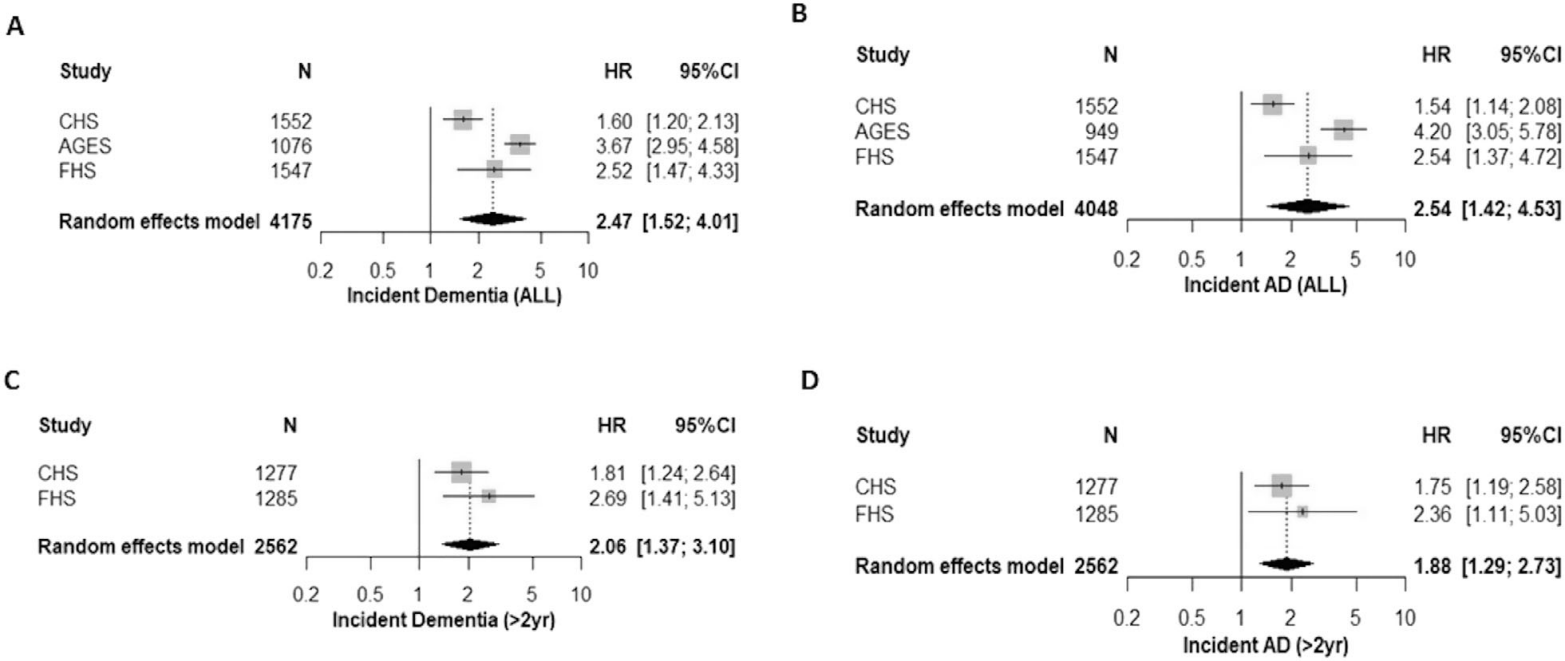
Pooled associations between circulating GFAP and incident dementia. Results are per unit increase in the standardized natural log of GFAP examining associations with (A) incident all cause dementia, (B) incident Alzheimer's disease dementia, (C) incident all cause dementia and Alzheimer's disease dementia (D) with dementia surveillance beginning 2 years after the blood draw for GFAP. Cox proportional hazard models over a maximum of 15‐year follow‐up with age as the time‐scale and adjustment for sex, education, race, diabetes, systolic blood pressure, antihypertensive medication use, body mass index, apolipoprotein ε4 status (at least one ε4 allele vs. none), and site (if a multi‐site study).

Cohort demographics of the sample included in the incident dementia analyses are provided in Table 3. Over the follow‐up period (15 years maximum), the percentage of incident dementia cases ranged from 4% to 21%. In each of the three cohorts with incident dementia data, higher blood‐derived GFAP levels were associated with increased risk of incident all‐cause (Fig. 3A) and probable AD dementia (Fig. 3B).

**Table 3:** Cohort demographics – incident dementia sample.

	FHS *N* = 1547	CHS *N* = 1552	AGES *N* = 1076
Age, years, mean (range)	73 (60–96)	77 (69–96)	76 (66–93)
Female, *n* (%)	859 (56%)	929 (60%)	610 (56%)
Education, *n*, %			
Less than high school degree	54 (3%)	302 (19%)	247 (23%)
High school degree	362 (23%)	449 (29%)	528 (49%)
Some college	467 (30%)	392 (25%)	179 (16%)
College degree or higher	664 (43%)	409 (26%)	129 (12%)
Race			
Black, *n* (%)	54 (3.5%)	189 (12%)	0 (0%)
White, *n* (%)	1415 (91.5%)	1356 (87%)	1076 (100%)
Other, *n* (%)	78 (5.0%)	7 (<1%)	0 (0%)
Body mass index, m/kg^2^	28 ± 5	27 ± 4	27 ± 4
Systolic blood pressure, mmHg	127 ± 16	136 ± 20	142 ± 20
Antihypertensive medication, *n* (%)	924 (60%)	798 (51%)	690 (64%)
Diabetes, *n* (%)	288 (19%)	38 (2%)	111 (10%)
Presence of APOE ε4 Allele, No. (%)	347 (22%)	368 (24%)	289 (27%)
Blood‐derived GFAP levels, pg/mL, median (Q1–Q3)	181 (131, 258)	245 (187, 341)	177 (130, 232)
Incident dementia, *N* cases/total *N* cases, (%)			
All‐cause dementia	56/1547 (4%)	271/1552 (17%)	227/1076 (21%)
Alzheimer's disease	43/1547 (3%)	247/1552 (16%)	100/949 (10%)
Incident dementia, diagnosed >2 Years After Blood Draw for GFAP, *N* cases/total *N* cases, (%)			
All‐cause dementia	40/1285 (3%)	166/1227 (13%)	–
Alzheimer's disease	30/1285 (2%)	159/1227 (12%)	–
Average time to dementia diagnosis, years	4.8 ± 2.3	3.5 ± 2.9	8.7 ± 3.6

All values represent mean ± standard deviation unless otherwise noted. AGES, Age, Gene/Environment Susceptibility, N/A; APOE, Apolipoprotein E; CHS, Cardiovascular Health Study; FHS, Framingham Heart Study; GFAP, Glial fibrillary acidic protein; Q, Quartile.

Meta‐analyses indicated that each SDU increase in log‐transformed blood‐derived GFAP was associated with an approximate 2.5‐fold higher risk for incident all‐cause (HR = 2.47 [95% CI: 1.52–4.01], *p* < 0.001) and AD dementia (HR = 2.54 [95% CI: 1.42–4.53], *p* = 0.002). A high level of heterogeneity was detected across cohorts (all‐cause dementia: *I* ^2^ = 0.90, *p* < 0.001, AD: *I* ^2^ = 0.90, *p* < 0.001). After censoring the first 2 years of follow‐up, the meta‐analysis of study level estimates from the FHS and CHS indicated significant associations between baseline GFAP levels with incident all‐cause dementia (Fig. 3C HR = 2.06 [95% CI: 1.37–3.10], *p* < 0.001) and AD (Fig. 3D HR = 1.88 [95% CI: 1.29–2.73], *p* < 0.001). Results across the two cohorts were homogenous (all‐cause dementia: *I* ^2^ = 0.07, *p* = 0.30, AD: *I* ^2^ < 0.001, *p* = 0.49).

### Circulating GFAP, cognition, neuroimaging outcomes, and incident all‐cause and AD dementia with stratification by race

Race stratified analyses conducted in CHS and CARDIA generally indicated similar results among Black and White adults (Table 4). Across races in CARDIA and Black adults in CHS, there were no significant associations between GFAP and general cognition. The association between GFAP and poorer global cognition reached statistical significance in White adults in CHS. Across races and cohorts, there were no significant associations between GFAP with total brain or hippocampal volume. Hazard ratios for incident all‐cause and Alzheimer's disease dementia were similar in Black and White adults in CHS. However, the findings were only statistically significant in White adults, which is likely attributable to the larger sample size.

**Table 4:** Associations between circulating glial fibrillary acidic protein with cognition, neuroimaging outcomes, and incident dementia stratified by race.

	Cohort	Black adults		White adults	
General cognition	CHS	*N* = 167	*β* = 0.008, SE = 0.20, *p* = 0.97	*N* = 1212	*β* = −0.162, SE = 0.06, *p* = 0.005*
	CARDIA	*N* = 241	*β* = −0.13, SE = 0.13, *p* = 0.33	*N* = 382	*β* = −0.10, SE = 0.12, *p* = 0.40
Total brain volume	CHS	*N* = 58	*β* = 2.963, SE = 2.03, *p* = 0.15	*N* = 541	*β* = 0.051, SE = 0.32, *p* = 0.87
	CARDIA	*N* = 244	*β* = −0.28, SE = 0.41, *p* = 0.50	*N* = 386	*β* = −0.05, SE = 0.33, *p* = 0.88
Hippocampal volume	CHS	*N* = 58	*β* = 0.046, SE = 0.032, *p* = 0.16	*N* = 541	*β* = −0.004, SE = 0.006, *p* = 0.45
	CARDIA	*N* = 244	*β* = −0.003, SE = 0.008, *p* = 0.76	*N* = 386	*β* = −0.005, SE = 0.006, *p* = 0.38
Incident all‐cause dementia	CHS	*N* cases = 51, total *N* = 189	HR = 1.62, 95% CI = 0.71–3.69, *p* = 0.25	*N* cases = 220, total *N* = 1356	HR = 1.64, 95% CI = 1.21–2.23, *p* = 0.0015*
Incident Alzheimer's dementia	CHS	*N* cases = 47, total *N* = 189	HR = 1.32, 95% CI = 0.55–3.15, *p* = 0.53	*N* cases = 200, total *N* = 1356	HR = 1.61, 95% CI = 1.17–2.22, *p* = 0.0038*
Incident all‐cause dementia, >2 years after blood draw for GFAP	CHS	*N* cases = 29, total *N* = 189	HR = 2.01, 95% CI = 0.53–7.56, *p* = 0.3	*N* cases = 137, total *N* = 1356	HR = 1.88, 95% CI = 1.25–2.83, *p* = 0.0023*
Incident Alzheimer's dementia, >2 years after blood draw for GFAP	CHS	*N* cases = 28, total *N* = 189	HR = 1.84, 95% CI = 0.47–7.24, *p* = 0.38	*N* cases = 131, total *N* = 1356	HR = 1.82, 95% CI = 1.20–2.77, *p* = 0.0049*

* *p*‐value < 0.05.

AGES, Age; CARDIA, Coronary Artery Risk Development in Young Adults Study; CHS, Cardiovascular Health Study; CI, 95% Confidence Interval; GFAP, Glial Fibrillary Acidic Protein; HR, Hazard Ratio, 95%.

## Discussion

The present study examined GFAP, a putative blood‐derived marker for ADRD, among participants free of prevalent dementia at baseline across four longitudinal community‐based cohorts. We examined multiple outcomes relevant for ADRD including general cognition, total brain volume, hippocampal volume, and incident all‐cause and AD dementia. The meta‐analysis conducted across cohorts indicated that higher circulating GFAP was associated with poorer general cognition, but not with total brain or hippocampal volume. In addition, elevations in baseline GFAP levels were strongly associated with increased risk of incident dementia over the up to 15‐year follow‐up period. Specifically, an SDU increase in logarithm transformed blood‐derived GFAP levels was associated with an approximate 2.5‐fold higher risk of incident all‐cause and AD dementia. To reduce the possibility of reverse causation, we repeated the analyses with dementia surveillance beginning at least 2 years after the blood draw for GFAP, finding consistent associations. Stratified analyses generally indicated similar associations between circulating GFAP with cognition, total and hippocampal brain volume, and incident dementia between Black and White adults; however, the study was underpowered to fully examine race‐specific outcomes. Overall, the results provide supportive evidence for the potential value of blood‐derived GFAP as a prognostic marker for incident dementia risk in population‐based settings, which may have use for aiding stratification in clinical trials targeting the preclinical disease stage.

Consistent with our findings, several prior studies have reported negative associations between blood‐derived GFAP levels and cognition.^18^, ^19^, ^20^, ^22^ A cross‐sectional study of 1843 Hispanic and non‐Hispanic white participants across the continuum of cognitively intact to AD dementia reported that higher serum GFAP levels were associated with poorer global cognition, learning, and memory.^19^ A separate study conducted in 114 older adults with unimpaired cognition, mild cognitive impairment, or AD reported that plasma GFAP levels explained 25% of the variance in memory, as well 10%–15% of the variance in visuospatial, language/semantic knowledge, and executive function domains.^20^ In our study, we found that a one SDU increase in the log‐transformed value of GFAP was associated with a 0.09 decrease in standardized general cognition units. However, the association between circulating GFAP and poorer general cognition only reached statistical significance in one of the four individually examined cohorts, despite consistency in the directionality of the effect. To enable comparison across studies with diverse batteries, cognition was examined using a composite general score. Evaluation of specific cognitive tests or domains may provide additional insight into the relationship between GFAP and cognitive function. A prior study of cognitively intact adults with and without elevated amyloid PET burden reported that blood‐derived GFAP levels were negatively associated with working memory and executive function, but not with verbal, visual, or episodic memory or global cognition.^21^ In addition, unlike many previous studies,^19^, ^20^, ^22^, ^24^ our sample was derived from population‐based cohorts and did not include individuals with dementia at baseline. The association between cognition and blood‐derived GFAP levels may be smaller in the context of normal aging and increase more saliently among those with an underlying neurodegenerative disease process. In support of this hypothesis, a prior study of cognitively intact older adults reported that circulating GFAP levels increased linearly with higher preclinical AD burden as assessed by amyloid PET.^2^

Across the four individual cohorts examined, as well as within the meta‐analysis, circulating GFAP levels were not significantly associated with total brain or hippocampal volume. Similar to our results, a prior population‐based study of 1327 older adults did not find any cross‐sectional associations between blood‐derived GFAP and MRI outcomes, including total brain volume, hippocampal volume, and cortical thickness.^18^ Interestingly, individuals with a five‐fold higher concentration of GFAP at baseline displayed accelerated hippocampal atrophy and decreases in cortical thickness over the up to 16‐year follow‐up period, suggesting that elevations in GFAP may predict future risk of neurodegeneration. Additionally, a previous study reported higher blood‐derived GFAP levels in cognitively intact older adults relative to those with MCI when matched for cerebral amyloid PET burden, leading the authors to suggest that GFAP levels may increase prior to frank neurodegeneration.^2^ Future longitudinal studies in population‐based cohorts will be necessary to further evaluate circulating GFAP as a prognostic indicator for neurodegeneration.

Results of the meta‐analysis indicated that each one SDU increase in log‐transformed GFAP was associated with an approximate 2.5‐fold higher risk of incident dementia. The pattern of results remained unchanged when dementia surveillance was delayed for at least 2 years following the blood draw for GFAP, further suggesting that elevations in the biomarker may precede dementia onset. Similar to our findings, a prior population study reported that five‐fold higher concentrations of plasma GFAP were associated with a 3.2 higher odds ratio of developing AD dementia four to 8 years later.^18^ Our findings extend the literature by examining a continuous range of blood GFAP values across three population‐based cohorts with an up to 15‐year surveillance period. Additionally, our study examined both all‐cause and AD dementia, and found similar associations. Of note, most dementia cases in our population‐based cohorts were attributed to AD and pathological confirmation was lacking, limiting our ability to assess differential associations across dementia subtypes. However, prior studies have reported elevations in blood‐derived GFAP levels across multiple dementia subtypes including vascular dementia, frontotemporal dementia, Parkinson's disease, and Creutzfeldt‐Jakob disease.^17^ Therefore, elevations in circulating GFAP are unlikely to be a specific marker for incident AD but may rather broadly indicate increased risk for neurodegenerative disease regardless of etiological cause.

GFAP is an intermediate filament protein found in astrocytes, the most abundant cell type in the brain.^15^ Astrocytes exert pluripotent effects in the central nervous system, modulating cellular proliferation, neuronal trophic factor secretion, blood brain barrier integrity, and response to injury.^45^ In the presence of oxidative stress, astrocytes convert to a reactive state with higher expression of GFAP.^11^ In individuals with AD, GFAP expression has been found to co‐localize with plaques and neurofibrillary tangles.^10^, ^12^ In addition, blood‐derived levels of GFAP have been shown to correlate with cerebral amyloid beta and tau retention assessed in vivo with PET imaging.^2^, ^21^, ^22^, ^23^, ^24^ A recent study reported that plasma GFAP levels predicted conversion to amyloid positivity,^22^ suggesting that elevations in GFAP may occur early in the disease process. These results are corroborated by our findings indicating that elevated circulating GFAP is associated with incident dementia risk over an up to 15‐year follow‐up period.

Our study has several strengths including the use of large, well‐characterized, longitudinal cohorts, inclusion of multiple outcomes relevant to ADRD, statistical adjustment for numerous potential confounds, extension of previous findings to community‐based samples, and assessment of GFAP using a standardized assay platform across cohorts. However, the results of the study must also be considered within the context of the study limitations. First, the overall sample was significantly more homogenous than the broader United States population. Within the two cohorts with higher representation of Black adults, CHS and CARDIA, stratified analyses were performed by race, which generally indicated similar outcomes across groups. However, there were fewer Black participants than White participants and the sample size did not permit stratified analyses for other ethnic and racial groups. As such, there remains a critical need for further validation of GFAP in diverse cohorts.^19^ While our study examined multiple ADRD endophenotypes including cognition, brain volumetry, and incident dementia, it lacks CSF and PET imaging biomarkers. Therefore, ascertainment of dementia was derived from clinical diagnostic criteria rather than biological characterization,^1^ which may contribute to heterogeneity in the findings. In addition, the cohort studies included in the meta‐analysis incorporated different cognitive assessments, MRI scanners, and neuroimaging sequences, which may also increase variability. However, we have successfully harmonized these outcomes in prior studies,^26^, ^27^, ^38^, ^46^ and the results of the meta‐analyses generally demonstrated homogeneity in outcomes across cohorts. Moreover, the ability to aggregate and interpret data collected using varied samples and cognitive and neuroimaging measures is crucial for wider scale implementation. In addition, while our study included up to 15 years of follow‐up data on incident dementia status, circulating GFAP levels were only evaluated cross‐sectionally. Future studies with circulating GFAP levels assessed across multiple timepoints will be important for determining the timeline and prognostic value of interval increases in these protein levels. Finally, another potential study limitation is that GFAP was assayed from both plasma and serum blood samples across multiple cohorts and institutions with varied storage times. While circulating GFAP levels have been shown to be stable across different blood components and freeze–thaw cycles,^25^ these factors may contribute to variability in results between cohorts.

In summary, our meta‐analysis of population‐based cohort studies indicated that higher blood‐derived GFAP levels were associated with poorer general cognition. More notably, each one SDU increase in log‐transformed circulating GFAP levels was associated with an approximate 2.5‐fold higher risk of all‐cause and AD dementia over the up to 15‐year follow‐up period. The association persisted dementia outcomes were assessed at least 2 years after the blood draw for GFAP. Overall, the findings suggest that elevations in circulating GFAP may occur early in the neurodegenerative disease process, highlighting the potential utility of the biomarker for aiding dementia risk prediction and improving stratification in clinical trials targeting the preclinical disease stage.

## FHS

This work was made possible by grants from the Alzheimer's Drug Discovery Foundation (GDAPB‐202010‐2020940), National Institutes of Health (N01‐HC‐25195, HHSN268201500001I, 75N92019D00031) and the National Institute on Aging (AG059421, AG054076, AG049607, AG033090, AG066524, NS017950, P30AG066546, UF1NS125513).

## CHS

This research was supported by contracts HHSN268201200036C, HHSN268200800007C, HHSN268201800001C, N01HC55222, N01HC85079, N01HC85080, N01HC85081, N01HC85082, N01HC85083, N01HC85086, N01HC15103, 75N92021D00006, and grants R01AG15928, R01AG20098, U01HL080295 and U01HL130114 from the National Heart, Lung, and Blood Institute (NHLBI), with additional contribution from the National Institute of Neurological Disorders and Stroke (NINDS). Additional support was provided by R01AG053325, K24AG065525, and R01AG023629 from the National Institute on Aging (NIA). A full list of principal CHS investigators and institutions can be found at CHS‐NHLBI.org.

## CARDIA

The Coronary Artery Risk Development in Young Adults Study (CARDIA) is supported by contract Nos. HHSN26820180003I, HHSN26820180004I, HHSN26820180005I, HHSN26820180006I, and HHSN26820180007I from the National Heart, Lung, and Blood Institute (NHLBI), the Intramural Research Program of the National Institute on Aging (NIA), and an intra‐agency agreement between NIA and NHLBI (No. AG0005).

## AGES

The Age, Gene/Environment Susceptibility‐Reykjavik Study was supported by NIH contracts N01‐AG‐1‐2100 and HHSN27120120022C, the NIA Intramural Research Program, Hjartavernd (the Icelandic Heart Association), and the Althingi (the Icelandic Parliament).

## Conflicts of Interest

Dr Bryan is on the board of directors and owns stock in GalileoCDS, INC. Dr. Gonzales and her husband own stock in Abbvie. Dr Nasrallah has received honoria from Biogen and Eisai. Dr Pase has received honoria from Flordis. Dr Seshadri has received consulting fees from Biogen. Dr Yaffe is a board member of Alector. All other authors report no relevant conflicts of interest.

## Author Contributions

The following authors contributed to (1). The conception and design of the study: MMG; JCB, JJH, TFK, MPP, HJA, ASB, LJL, MF, RPT, SS, CLS; (2) Acquisition and analysis of data: CW, CPW, QL, JCB, ZL, JJH, SG, EAT, DMP, LD, KJM, BMP, WTL, THM, VG, DM, SMG, KY, SS, RNB, IMN, CSD, ASB, LJL, MF, RPT; (3) Drafting a significant portion of the manuscript or figures: MMG, CW.
